# Promoting social responsibility amongst health care users: medical tourists’ perspectives on an information sheet regarding ethical concerns in medical tourism

**DOI:** 10.1186/1747-5341-8-19

**Published:** 2013-12-06

**Authors:** Krystyna Adams, Jeremy Snyder, Valorie A Crooks, Rory Johnston

**Affiliations:** 1 Faculty of Health Sciences, Simon Fraser University, Blusson Hall, 8888 University Drive, Burnaby, British Columbia, Canada; 2 Department of Geography, Simon Fraser University, Robert C. Brown Building, 8888 University Drive, Burnaby, British Columbia, Canada

## Abstract

**Background:**

Medical tourists, persons that travel across international borders with the intention to access non-emergency medical care, may not be adequately informed of safety and ethical concerns related to the practice of medical tourism. Researchers indicate that the sources of information frequently used by medical tourists during their decision-making process may be biased and/or lack comprehensive information regarding individual safety and treatment outcomes, as well as potential impacts of the medical tourism industry on third parties. This paper explores the feedback from former Canadian medical tourists regarding the use of an information sheet to address this knowledge gap and raise awareness of the safety and ethical concerns related to medical tourism.

**Results:**

According to feedback provided in interviews with former Canadian medical tourists, the majority of participants responded positively to the information sheet and indicated that this document prompted them to engage in further consideration of these issues. Participants indicated some frustration after reading the information sheet regarding a lack of know-how in terms of learning more about the concerns discussed in the document and changing their decision-making. This frustration was due to participants’ desperation for medical care, a topic which participants frequently discussed regarding ethical concerns related to health care provision.

**Conclusions:**

The overall perceptions of former medical tourists indicate that an information sheet may promote further consideration of ethical concerns of medical tourism. However, given that these interviews were performed with former medical tourists, it remains unknown whether such a document might impact upon the decision-making of prospective medical tourists. Furthermore, participants indicated a need for an additional tool such as a website for continued discussion about these concerns. As such, along with dissemination of the information sheet, future research implications should include the development of a website for ongoing discussion that could contribute to a raised awareness of these concerns and potentially increase social responsibility in the medical tourism industry.

## Background

Medical tourism, the practice of patients traveling out of country with the intention to receive medical care paid for out of pocket, is considered an expanding industry globally [1,2]. While there is a dearth of empirical evidence on patient flows and the impacts of medical tourism, media coverage often provides anecdotal stories that indicate the potential for surgical complications, and poor quality or unnecessary care due to a lack of regulation in the industry [3]. Partially due to the lack of empirical evidence available about medical tourism, researchers are concerned about the gaps in the information medical tourists may be accessing regarding potential impacts of medical tourism on both individual safety and health equity [4,5].

Medical tourism is receiving increasing attention, both as a developing industry and a topic of research interest [6]. While there appears to be a wide variety of medical tourism flows, including travel between both countries of similar and different levels of economic development, information available to medical tourists demonstrates a concerted effort on behalf of low and middle income countries to entice persons to travel from developed to developing countries for medical care [7,8]. This recent trend of travel from more developed to developing countries has become a focus for research on the impacts of medical tourism on global health equity [9]. Discussions by researchers about growing health inequities globally have identified the growth of the medical tourism industry as potentially one cause of these inequities [10]. The rise of neoliberalism and economic globalization in the past few decades has resulted in health care being increasingly conceptualized as a tradable commodity across borders. The medical tourism industry markets to patients as consumers of health care, encouraging individuals to take responsibility for their health by taking advantage of potential cost savings of medical treatment in developing countries and high quality care available in private facilities catering to medical tourists [11]. Patients may be motivated to travel to access procedures which are domestically unavailable or require undesired waiting times, receive care and recover in a relaxing or exotic landscape, and/or reduce costs for procedures domestically paid for out-of-pocket [7].

Research indicates that many Canadian medical tourists use word-of-mouth or the internet to inform their decisions regarding this practice, which may result in decision-making that is uninformed or biased around individual safety and treatment outcomes [12]. Canadian medical tourists may also be unaware of ethical concerns regarding potential impacts of medical tourism on global health equity, particularly potential impacts to destination and departure country health resources and health care systems [13,14]. While researchers are increasingly discussing these potential ethical concerns related to medical tourism, medical tourists may be unable to act in a socially responsible manner if uninformed of these impacts [15].

Here we explore the responses of Canadian medical tourists to an information tool intended to encourage more informed and ethical decision-making around medical tourism. This tool was developed through an iterative process that drew on values from guidelines in related domains as well as public health communication research to effectively inform Canadians about ethical concerns related to medical tourism. Through analyzing feedback received in one-on-one interviews with former Canadian medical tourists, in this paper we examine the potential usefulness and impacts of such a tool on patients’ decision-making, as well as the contexts in which such a tool might better inform and promote social responsibility amongst Canadian medical tourists and ultimately a more ethical medical tourism. Furthermore, this research provides insight into considerations for the field of public health in promoting social responsibility in the provision and utilization of health care.

### Ethical issues in medical tourism and the need for enhanced awareness

Despite a lack of empirical evidence on the impacts of medical tourism, researchers have indicated several ethical concerns relating to medical tourism’s potential impact on both an individual and societal level within destination and departure countries, as well as on a global scale [13,14]. Medical tourism may negatively impact upon individual health due to a lack of risk communication to patients and/or inadequate informed consent [2,14]. Furthermore, the medical tourism industry may divert resources from the public to private sector. Increasing privatization of health care may reorient priorities in the provision of health care to provide more profitable specialized care, including care which meets the needs of foreign patients travelling for some form of specialized care [14,16]. This reorientation of health care may reduce access to desired health care for local patients in medical tourism destination communities [1]. In countries of origin for medical tourists, potential complications affiliated with procedures obtained abroad, particularly due to a lack of regulation in the industry, may divert resources to treat these complications. Furthermore, reduced pressure for system reform if patients leave the health care system to seek out care abroad may result in only those persons able to travel as a medical tourist accessing health care [1,13]. Finally, increased movement of patients across borders to access medical care may increase the transmission of infectious diseases [17]. Concerns for medical tourism’s negative impacts on health outcomes and health equity indicate a burden of responsibility for those engaging in medical tourism to consider these impacts and act in a socially responsible manner when providing and utilizing health care [14].

The primary ethical concerns surrounding the development of medical tourism and its impacts are also related to larger impacts on global health equity. While countries that are promoting themselves as medical tourism destinations cite economic motivations for industry growth [18], these economic advantages may come at the cost of undermining initiatives that intend to improve health equity both within and between nations. Increases in privatized health care globally have detracted from health care system strengthening required to meet the goals of “Health for All” outlined in the World Health Organization’s Declaration of Alma Ata [19] and protect the human right to health [20]. While some medical tourism stakeholders have mentioned potential benefits of the practice to health outcomes and health equity, including improved quality control due to international accreditation of medical tourism facilities, increased training opportunities for health professionals treating foreign patients, and health worker retention in places experiencing high levels of brain drain to other countries, lack of regulation by some destination governments and the industry itself may neglect considerations for health outcomes and health equity in stakeholder decision-making [9].

Ethical concerns for medical tourists’ destination and departure countries are related to the provision of health care within national borders. For destination countries, concerns about the growth of the medical tourism sector are related to the impacts of medical tourism on public health care due to the potential growth of the private sector [8]. The promotion of medical care to foreign patients may encourage a shift in health resource allocation resulting in a potential brain drain of health human resources from the public to the private sector and the diversion of public resources such as land or public finances to medical tourism businesses [21]. Medical tourism may incentivize training of health workers for more curative, costly and complicated procedures that are appealing to medical tourists [7]. This emphasis on more curative care may result in neglect for the provision of appropriate primary health care to local populations and an increasing normalization of privatized health care [1]. For departure countries, medical tourism raises concerns for these countries’ health systems due to the potential diversion of resources to treat medical complications resulting from patients engaging in medical tourism [14]. Furthermore, medical tourism raises concerns for health equity within nations if only patients willing and able to travel for medical care are able to access certain medical procedures [1].

Ethical concerns regarding patient health in medical tourism are particularly focused on the lack of neutral information provided to medical tourists regarding risks and safety concerns and the implications of this for patients’ abilities to achieve informed consent. These safety concerns include potential medical complications related to the procedure itself, potential medical complications from travelling following a medical procedure, and/or inadequate continuity of care after returning home [17]. Patients may be unaware of these safety concerns due to lacking familiarity with the destination health care system, language or cultural barriers to adequate communication between medical tourists and health care workers, and/or lacking transparency regarding quality of medical facilities or health care workers [2].

Medical tourists and stakeholders in the medical tourism industry may be unaware of the ethical concerns outlined above, and as such, are unable to act in a socially responsible manner [14]. This lack of awareness may be due to biased sources of information, including sources with a vested interest in the profitability of the medical tourism industry [22]. Socially responsible engagement in the medical tourism industry requires individuals to act in a manner that respects the inherent dignity of all other humans [23]. Individuals are thus responsible for encouraging the development of social sustainability, defined as the creation of conditions that enable people to lead lives of personal value [24]. Improved health outcomes and health equity are increasingly recognized as cornerstones of social sustainability [25]. As such, the provision and utilization of health care contributes to social sustainability and necessitates responsible allocation and use of health resources in such a manner that optimizes health outcomes and health equity [26], even in relation to the private medical tourism industry. We contend that creating awareness of the ethical concerns of medical tourism will encourage socially responsible actions by individuals engaging in medical tourism, stakeholders in the medical tourism industry, and policy makers developing regulations for the industry [3]. It is for this reason that we have made an informational tool for Canadians considering medical tourism that prompts consideration of ethical, equity, and safety issues alike.

Informing individuals such as medical tourists about health issues can be done most effectively following extensive formative research to better understand the target audience and determine the ideal means of providing the information [27]. Thus, in the remainder of this article we present the findings of the formative, qualitative research we conducted in order to inform the development of an information tool for Canadians considering medical tourism that prompts consideration of ethical, equity, and safety issues. The process of formative research is useful for tailoring message points and determining effective dissemination of the information. Furthermore, formative research helps identify gatekeepers to the primary audience who either encourage or discourage information being provided to the primary audience [27]. Formative research activities such as focus groups and pre-testing of informational tools with primary target audiences and gatekeepers provide valuable insights that can be used for tool refinement and implementation [27,28]. With this in mind, in the sections that follow we examine feedback provided to us by intended users of the informational tool so as to assess the potential impact of such an information tool, inform revision and improvement upon the tool, and determine future steps for effective dissemination of the tool leading to more informed decision-making by Canadians considering medical tourism.

## Methods

**Figure 1:**
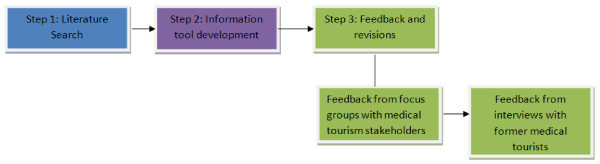
Multi-step process for information tool development.

To develop the information tool on which the current formative study is based, an iterative process was utilized. This multi-step process is summarized in Figure 1 below. Here we report exclusively on the findings from the interviews with former Canadian medical tourists in Step 3 of this process. Although this tool was developed using ethical values and principles as a way to enhance consideration of ethical issues during the decision-making process, words such as ‘ethics’ and ‘values’ were deliberately excluded from the text as our previous research has shown that medical tourists can respond negatively to such terms, interpreting them to be explicitly judgmental. Instead, ethical concerns, along with equity and safety concerns were shared throughout the tool without being framed as such. As a health communication tool with the objective of informing and not guiding individual action, the target audience includes all Canadians that may be in the pre-contemplation or contemplation stage of decision-making regarding accessing care out of Canada as a medical tourist [29]. While this tool may provide useful information to all Canadians regarding this growing industry, it is intended to encourage more informed decision-making when considering or planning to travel out of country for private medical care, and as such, this stage of formative research focused on gaining feedback through semi-structured interviews with Canadians that have already travelled as medical tourists.

### Recruitment

Former medical tourists were selected to participate in this formative research as both a convenient sample to access in comparison to Canadians that may be considering participating in medical tourism, as well as a rich source of information regarding the impact of such a tool during the decision-making process given their experience. Participants were recruited using the following channels simultaneously: 1) Craigslist advertisements posted on sites for all major Canadian cities; 2) advertisement in a Vancouver newspaper; 3) posting invitations to participate in online medical tourism forums; and 4) snowball sampling through interview and focus group participants’ networks. Information was provided for interested persons to contact either a toll-free phone number or email for further information and assessment of eligibility. Eligible potential participants were provided with a consent form to be read and signed before commencement of the interview. Participants were informed that prior to recruitment, the study was granted ethics approval from the Office of Research Ethics at Simon Fraser University.

Eligibility criteria for study required participants to be: 1) 18 years or older; 2) a holder of a provincial medical card; 3) someone who successfully pursued a surgical procedure outside of Canada that was neither a transplantation nor a reproductive surgery (as these procedures often involve third parties and raise distinct ethical issues); and 4) someone who paid privately for the surgery sought abroad and for whom the procedure was not performed based on a referral from a Canadian physician. Canadians who had travelled for transplantation or reproductive procedures were not included in this study due to the additional ethical concerns with these practices that are not covered in the information tool.

### Data collection

Twenty-four semi-structured interviews were completed between October 2012 and December 2012. We conducted as many interviews as possible during this two-month period, after which we planned to cease data collection. The interviews were all conducted over the phone and recorded for future transcription. The shortest interview lasted just over 20 minutes, with the longest lasting nearly an hour and a half. The majority of interviews lasted approximately 45 minutes. The range in interview times is due to the semi-structured approach which provided some questions to guide the discussion and ensure meaningful feedback, but allowed for freedom for the participants to speak openly, which may broaden the understanding of the topic at hand [30].

**Figure 2:**
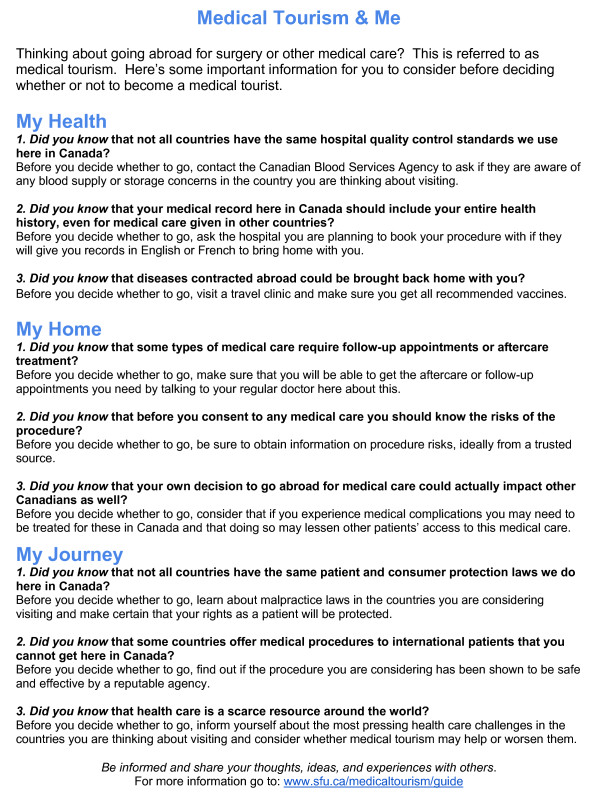
Draft information sheet.

All interviews were conducted by the first author using a semi-structured interview guide. The guide contained questions divided into four parts. The first part addressed basic background information of the participant including demographics, general health, and past travel experience. The second part asked questions relating to the participants’ general decision-making behaviour. The third section asked questions related to participant’s experiences as a medical tourist, particularly in terms of how they made the decisions relating to their trip. Both the second and third section probed the participant about the types of information they were considering during their decision-making process as a medical tourist. The final section of the interview guide contained questions asking for feedback on a draft version of the information sheet (see Figure 2), particularly the usefulness of such a document in the participants’ context and recommendations for improving the document.

### Data analysis

Interviews were transcribed verbatim. The first author reviewed all of the transcripts and extracted basic demographic information, basic procedure and trip information, as well as general feedback (positive or negative) on the information tool for each participant into a spreadsheet. She then assigned 6 transcripts for independent review by each team member. Three of these transcripts were the same across all members while three were unique. This strategy was used to minimize redundancy in transcript review and ensure that all members would encounter the range of opinions shared by participants. Following transcript review, we met to discuss emerging themes in participant feedback on the tool. Given the practical or applied nature of this formative study, the themes identified were primarily a priori or deductive in nature in that they focused on tangible improvements to specific aspects of the informational tool. After these themes were identified, the first author hand reviewed the transcripts and extracted segments that pertained to each into a Word document organized under six headings: positive feedback, negative feedback, specific recommendations, impacts of the tool on decision-making, potential places to access such an information tool, and type/tone of information conveyed in the tool. Verbal and written consent was obtained from interview participants for the use of anonymized quotes in published materials.

## Results

Overall, the 24 interview participants ranged in age from 24 to 65. Nine participants identified themselves as male and the remainder as female. Fifteen of the participants had traveled out of Canada for the Chronic Cerebrospinal Venous Insufficiency (CCSVI) procedure, also called renal or jugular angioplasty by participants. The remaining nine participants accessed a lumbar disc replacement surgery, a dental surgery, a gastric band surgery, a tattoo removal through surgical incision, a vertical sleeve gastrectomy, an eye-lid surgery, a meniscus surgery, and a cholecystectomy. Five of the 24 participants indicated that they had existing family connections in the destination where they underwent the medical procedures, with four of these five participants having emigrated to Canada from that country. When examining destination choice amongst CCSVI patients, seven out of fifteen participants had traveled to the United States, with the remainder traveling to Bulgaria, Poland, Mexico, India, Germany, and Egypt. Non-CCSVI patients traveled to Egypt, Germany, Spain, Philippines, Venezuela, Thailand, Hong Kong, Romania, Mexico and India for surgery. Overall, five participants had participated in multiple medical tourism trips, with four out of five repeating the CCSVI procedure in a different location, and one travelling for three separate medical procedures in three different locations.

The 24 participants all read the information sheet prior to commencing the interview to allow for discussion on this document during the interview. The feedback provided by these participants is described in the remainder of this section. This feedback indicates the perceptions of these persons on the information sheet itself, with a particular focus on the format, usefulness and potential influence of the document on medical tourists’ decision-making.

### Feedback on format

Overall, almost all participants were generally positive when discussing the format of the tool. Regardless of the procedure sought or the country visited, participants had similar feedback on the format of the information tool, which was overwhelmingly positive. Positive feedback relating to format indicates that participants appreciated the concision of the document, including its short sentences and “*conversational*” language. Participants indicated that the document is “*easy to read*”, relating this ease to both layout and language. Feedback on the format specifically indicates that the language is “*very clear*” and “*basic*”. This was particularly important because many participants commented that the language and level of detail contained in the document must be easily accessible to persons with “*standard education*” and “*standard English speaking”* abilities. Furthermore, participants indicated that the short one-page format is favourable as the limited text is “*not boring*”. The question-style format of the document was well-received by some participants who found this style encouraged persons to reflect on the information and take this information to do further research on their own, while others preferred that it be populated entirely with standard sentences.

While there were very few negative comments about the format of the document in terms of layout, participants provided formatting suggestions to improve the appeal of the document. Some participants recommended more of a checklist format over the question-style format. This format was recommended to avoid the document being “*wordy and plain*”. Several participants also indicated that some of the repetition in wording was not appealing and made the document seem repetitive and condescending. The most common recommendation for improving the format of the document was to include pictures, images or more colours to increase the visual appeal.

In terms of language, while all participants agreed that the language was easy to understand, some participants pointed out areas where the information provided did not lend itself to specific actions. These participants demonstrated frustration with being unsure what the information in the tool was trying to convey. One participant indicated that education level would really impact whether a “*person’s going to give it a second thought or not*” and suggested that while the language may be clear, the intended information may not be fully understood by all persons. Furthermore, some participants suggested that readers may have made up their mind before accessing the tool and may not have an open mind when reading the document, indicating challenges in conveying intended information through the format of an information tool.

Another suggestion by participants for maintaining the concision of the document while providing useful information was the inclusion of small anecdotes or stories as part of the format would be useful. Participants indicated that describing personal experiences through anecdotes helps empathize with the reader by creating a “*personal touch*”. They felt that a format with anecdotes illustrating both positive and negative experiences would prompt persons to do further research while creating a neutral tone.

### Feedback on usefulness

Positive feedback on the usefulness of the information in the tool was fairly congruent, with similar feedback coming from participants who had travelled to different countries for distinct procedures. In general, participants provided positive feedback on the usefulness of the document in prompting further research by those considering medical tourism. Many participants stressed the importance of medical tourists doing “*as much research as you can*” after reading the document and felt that this document is successful in “*giv[ing] you things to think about*” while engaging in this research. Some participants indicated specific examples of points of information from the document that they had not previously considered that encouraged serious contemplation when reading the tool. Participants that indicated the tool would be useful for promoting further contemplation and research of medical tourism generally felt that the information was relevant to anyone travelling out of country for medical care, regardless of procedure.

Only a small minority of participants indicated that they did not think the informational tool would be useful for Canadians considering medical tourism. There were two main bases for this perspective: 1) even if the document does prompt a reader to do further research, it does not provide enough guidance or insight into how to do this research, or 2) the information presented in the document is not relevant to their experience. Regarding the first basis, some participants indicated frustration after reading this document and wanting to know more about the issues being raised but not being sure of where to turn to access it. While the tool does provide a link to our own research website at the bottom of the page, some participants would have liked to have seen links to sources of information for each point. Furthermore, some participants, particularly CCSVI patients, were unsure what to do if they wanted to go abroad for surgery that they were denied access to domestically in that they felt this circumstance raised unique issues. Overall, participants who were unsure as to how to do further research after reading the document felt that the tool was “*unrealistic*” as it lacked the sufficient amount of information necessary to make informed decisions regarding the points raised in the tool.

Participants who did not find the tool particularly relevant indicated that while they feel it is important to do a great deal of research before travelling as a medical tourist, often the motivations for travelling involve some level of desperation which really impacts upon the decision-making process. In other words, it was thought that this search for hope or push abroad out of desperation impacts the usefulness of the tool in that this reality far outweighs the importance of any of the prompts shared in the document. This desperation for medical treatment makes further research about the information contained in the document a “*bit of a tall order*”. Many participants said that at the time of deciding whether or not to travel out of Canada for medical care, they were in a lot of pain and this would have impacted their abilities to spend time searching for additional information to guide them in their decision-making both in terms of energy levels and because it would be “*something else to worry about*”.

Some participants did not agree with all of the potential negative impacts of medical tourism suggested in the tool. They were most critical of suggestions that local citizens may not benefit from medical tourism hospitals and clinics. These participants often cited witnessing locals being treated at the facilities they visited or the undeniable economic gain to the community as providing confidence that medical tourism does not negatively impact destination communities. One participant said that he or she was “*keeping the faith that us being there would provide more money*” into the local economy, indicating that she assumed an overall positive benefit of medical tourism on the local community. Likewise, some participants disagreed with points in the document related to potential negative impacts on Canada, indicating that leaving Canada for care would provide overall benefits such as “*reduce[d] waiting times*” and “*decrease[d] costs*”.

### Potential influence of the information tool on decision-making

When participants were asked whether or not the information tool would have impacted their decision-making, many indicated that they probably would have still travelled out of country for their medical procedure but that it would have prompted them into conducting more research before leaving Canada. They indicated that the document might specifically impact upon intended users’ expectations by encouraging further research, the outcome of which would provide more realistic expectations when considering or planning a medical tourism trip. On the other hand, some participants clearly stated that they would not have considered the information in this tool, either because they perceived that the document was implicitly and explicitly biased against medical tourism that would have turned them off from reading the tool, or because they had already considered all of the information contained in this document.

When asked directly about ethical prompts included in the tool, most participants said they were not apparent to them in their review of the document. One participant said that it did not raise any ethical concerns to him or her because the tool “*is just stating a fact*” and “*not [presenting medical tourism] as either pro or con”,* indicating that the perceived neutrality of the document impacted her or her perception on whether the information presents ethical concerns. The few participants that felt that the information in the tool raised ethical concerns did not see this tool as being explicit enough when raising these concerns, with one stating that “*the document doesn’t really bring out the ethical concern of monopolizing [health] resources*” and another saying “*I don’t see how this document raises those type of concerns; however, they’re [concerns about patients in destination countries accessing medical] very valid concerns… that could be an ethical issue. But, if you bring it back to the document, I don’t see how that’s really linked to the type of questioning you ask in the document*”.

## Discussion

The feedback provided by Canadians that had previously travelled for medical tourism demonstrate varied opinions regarding the format, usefulness, and potential impact of an information tool for Canadians considering engaging in medical tourism. Although there was not complete agreement amongst all participants in terms of their opinion of the tool, the findings shared above demonstrate recurring themes throughout the feedback. While feedback on tool format was overall quite positive, feedback on usefulness and impact of the tool was divided between a few different opinions that ranged from strongly positive to strongly negative. The positive feedback indicated that the concision of the tool and language accessibility were generally appealing to participants. Furthermore, the overall positive feedback on usefulness of the tool indicated that as a source of information that prompts further research and contemplation about medical tourism prior to coming to a final decision, this tool will be of interest to a wide range of persons considering medical tourism as it is not guiding any particular action. This positive feedback leaves us encouraged a broadly disseminated tool would be appropriate for the intended target audience and could result in increased awareness of ethical concerns of medical tourism.

Negative feedback about the tool identified potential barriers to increasing awareness of ethical concerns for medical tourism. First of all, strong negative feedback on the document focused on the perceived negative or biased tone of the document and indicated that this might dissuade people from considering the information presented in the document. However, we expect that participants may have been more likely to view the tone as negative given that they had already travelled as medical tourists and may be more likely to reject information indicating potential negative impacts of medical tourism or to feel defensive around the choice they made [12]. For example, many participants described in detail their experiences accessing high quality medical care as a medical tourist and this may have biased their view on the potential safety concerns related to this practice. Similarly, participants that disagreed with potential negative impacts on access to medical care for destination populations might have been impacted by their experiences with a particular community, which might not be the case for persons that have not already travelled as a medical tourist.

Negative feedback on the usefulness of the document did highlight a lack of ‘know-how’ for what to do next with the information provided in the tool. Even when participants acknowledged that this tool increased their awareness of potential impacts of medical tourism, many were unsure how this might translate to action. Participants experiencing chronic pain said that, given their desperation to access care, their focus on improving their individual quality of life would likely have prevented them from considering potential societal impacts had they read this document before travelling for care. These results are similar to previous studies that have found Canadian medical tourists are less inclined to consider potential societal impacts resulting from medical tourism when asked about ethical concerns, and more likely to discuss concerns about the Canadian health care system contributing to their decision to participate in medical tourism [14]. In fact, medical tourists are most likely to travel for medical care in response to their experiences of feeling ‘abandoned’ by their own health care system [31]. As the majority of participants in this study were diagnosed with multiple sclerosis and travelled outside of Canada to access an unavailable procedure, many participants’ likely experienced high levels of frustration trying to access care in the Canadian health care system before deciding to travel outside of Canada for the CCSVI procedure. The interviews demonstrated that the suggestion to contemplate potential ethical and health equity impacts of medical tourism on the Canadian health care system or health care systems globally may further frustrate patients that feel abandoned by the health care system and are desperate to access care. These findings agree with previous research which describes medical tourists as escaping the constraints of a national health care system by expanding their health care system to a global scale [31].

Participants mentioned several times that their desperation resulted in quick decision-making around medical tourism, which may limit informed consent, an existing problem in medical tourism [2]. This tool intends to encourage further consideration of information regarding potential impacts of medical tourism but, as demonstrated in the results, this may be frustrating for participants who feel as though they are in a desperate situation, face time constraints, and/or are unsure where to begin this research. However, we believe this frustration, if voiced, could potentially have a positive impact by promoting a need for greater research and regulatory structures within the medical tourism industry that facilitate more ethical decision-making and informed consent by medical tourists. Research on policy development indicates that shaping the public debate may have a large impact on resulting policy outcomes [32] and an information tool such as this one can definitely play a role in shaping the public debate around medical tourism in Canada and increasing advocacy surrounding ethical medical tourism and health service provision and utilization.

Following analysis of feedback from interview participants, the research team utilized this feedback to further revise the information provided in the document. Furthermore, we took into consideration feedback on document format from the interview participants as well as consultation with existing one-page health communication documents provided to patients in travel health clinics to guide the design of a document for dissemination. This final document can be seen in Figure 2. Dissemination of this document will take place through distributing copies to travel health clinics, primary health care providers, health authorities, and other groups interested in providing this document to persons using their services.

### Wider relevance

The results and discussion of feedback from interview participants provide insight into the challenges and useful considerations that researchers, policy makers, and health care providers may wish to contemplate when considering the communication of ethics in health care utilization and provision. Global health researchers have identified a need for the application of ethical frameworks and tools to public health to encourage more fair and just allocation of health resources globally [33,34]. Consumer guidelines utilize a format that has been effective at increasing public discussion about some of the existing ethical issues in practices such as tourism. This public discussion may serve to introduce and reinforce more ethical practices through increased awareness of one’s social responsibilities [35]. This research study demonstrates that guidelines or information sheets tailored to health care users may provide an effective means of engaging persons accessing health care into conversations and further contemplation about one’s social responsibilities when accessing health care. As such, this research may be of interest to persons interested in promoting more just and fair allocation of health resources.

### Limitations

This study is limited in its ability to develop an understanding of persons’ actions following the consideration of information provided in this document. As the methods utilized for this analysis involved one-time interviews with persons that had already travelled for medical tourism, these persons did not speak on any future actions that they might take. While this study explored contexts in which this information may not be considered by someone reading the document, for those that did consider this information useful to the decision-making process, this study is limited in its ability to provide insight into the actual use of this information in this decision-making process. As such, the ability for this study to understand the effectiveness of the information sheet is limited.

### Future directions

While the findings of this formative study suggest that an information sheet focused on ethical concerns with medical tourism can play a useful role in raising awareness about these concerns and advocating for more ethical practice in the industry regardless of procedure, more research is needed to further explore this potential given the limitations of this study. For example, feedback from persons that have not already travelled as medical tourists could provide additional insight on the potential impact of this information tool. Additionally, there was limited feedback provided by participants on their understanding or potential engagement with the information provided in the tool. While participants easily engaged in conversation about potential impacts of medical tourism in previous questions, when asked about their thoughts on whether the information in this tool presented them with any ethical concerns, the use of the word “ethics” seemed to limit participants’ engagement. This may be due to participants feeling uncomfortable discussing the ethics of an activity in which they have already engaged. This more limited discussion of ethics resulted in some lack of clarity regarding comprehension of the information presented in the tool. This suggests that future research with persons contemplating medical tourism should focus on greater in depth-discussion about the impacts of such a tool in terms of engagement with the information presented within. Furthermore, an additional resource such as a website could provide greater details, including anecdotes and alternative methods of describing the ethical concerns of medical tourism. The link to the website could be provided on the information sheet, and further research could explore feedback on the usefulness of such a website.

## Conclusions

This paper has explored the use of an information tool as a means of increasing individuals’ awareness of ethical concerns in medical tourism to enable Canadians to make more informed decisions about private health care utilization abroad. With existing sources of information for medical tourists commonly demonstrating a lack of neutral guidance [12,16], this information tool responds to a pressing, practical knowledge gap. According to feedback from interviews with former Canadian medical tourists, the tool we have developed has the potential to raise awareness of ethical concerns during the decision-making process. However, it is unknown at this point whether this awareness will lead to shifts in attitude and changes in behaviour at both the individual and societal level to contribute to social responsibility in health care provision and utilization, and ultimately improved global health equity [28]. Moving beyond the formative research presented herein, implementation of the tool and evaluation of its uptake and utilization will shed light on whether or not such awareness will lead to these types of shifts and changes.

## Competing interests

The authors declare that they have no competing interests.

## Authors’ contributions

KA conducted 23 of the interviews while JS conducted 1 interview. KA reviewed all interview transcripts and the remaining authors reviewed 6 transcripts each. All authors contributed to the development of the interview guide, discussions of emerging findings from the interviews, and revisions to the information sheet. KA wrote the manuscript with input from all other authors. JS and VAC reviewed and approved the final manuscript. All authors read and approved the final manuscript.
